# Predicted protein-protein interactions between sugar beet root maggot trypsins and sugar beet Kunitz trypsin inhibitors using deep learning

**DOI:** 10.1016/j.dib.2026.113020

**Published:** 2026-06-27

**Authors:** Yerang Lee, Sidra S. Rahim, Nadim W. Alkharouf, Chenggen Chu, Vincent P. Klink

**Affiliations:** a Department of Computer and Information Sciences, Towson University, Towson, MD, 21252, USA; b USDA-ARS-NA- Northern Great Plains Research Laboratory, 1307 N 18TH ST, Northern Crop Science Laboratory, Fargo, ND 58102, USA; c USDA-ARS-NEA-BARC, Molecular Plant Pathology Laboratory, Building 004, Room 122, BARC-West, 10300 Baltimore Ave., Beltsville, MD 20705, USA

**Keywords:** Plant pathogen, Insect, Pest, Diptera, Sugar beet root maggot, *Tetanops myopaeformis*, Sugar beet, *Beta vulgaris*, Stakeholder, Trypsin, Kunitz trypsin inhibitor

## Abstract

Sugar beet (*Beta vulgaris* ssp. vulgaris), SB, is one of two plants from which sugar is broadly extracted, annually accounting for 55% of U.S. sugar ($1B, U.S.), and 35% global raw sugar ($4.6B). The sugar beet cDNA DV501688, encoding a serine threonine protease inhibitor (*Bv*STI) was shown to have increased relative transcript abundance in sugar beet during a resistant reaction to the Dipteran sugar beet root maggot, *Tetanops myopaeformis* SBRM, pest. BLASTp searches of the sugar beet proteome identified 21 additional proteins, each having homology to Kunitz trypsin inhibitors (KTIs) with *Bv*STI annotated as *Bv*KTI1. Prior deep learning analyses predicted that *Bv*KTI1 bound each of the 9 *T. myopaeformis* trypsins in the predicted binding loop region located between β-strands 4 and 5 of KTI proteins. The identification of 21 additional *Bv*KTI proteins suggested the plant-insect pest interaction may be more complex, requiring further analysis. The deep learning analysis presented here, using AlphaFold, suggeststhe different *Bv*KTIs may not be equally effective in binding the *T. myopaeformis* trypsins. *Bv*KTI13 is predicted to bind all 9 *T. myopaeformis* trypsins with high confidence interface predicted template modelling (ipTM) scores between 0.77 and 0.82. In contrast, *Bv*KTI14, *Bv*KTI20, and *Bv*KTI22 are predicted to not bind any of them (ipTM scores < 0.6). The remaining *Bv*KTIs are predicted to have intermediate capabilities to bind *T. myopaeformis* trypsins, having predicted binding for some and lacking predicted binding for the others. However, the AlphaFold-based results are predictive and not scientific justification/evidence.

Specifications Table

**Table:** 

Subject	Omics
Specific subject area	Genomics
Data format	Raw, Analyzed, Filtered
Type of data	Table, Fig.s
Data collection	
Data source location	
Data accessibility	Repository name: NCBI; The Beta vulgaris Resource, Phytozome Direct URL to data: https://www.ncbi.nlm.nih.gov/bioproject/PRJNA1026092; https://bvseq.boku.ac.at/; https://phytozome-next.jgi.doe.gov/ The data has been deposited in Genbank SRA archive found at NCBI, or is available at the other sites, meeting the requirements for submission.

## 1 Value of the Data

The sequenced *T. myopaeformis*, sugar beet root maggot (SBRM) genome, TmSBRM_v1.0, provides data that researchers can use to understand its biology and insect pest nature. The value of the data is compounded when used in parallel with its host, *B. vulgaris* spp. Vulgaris of which there are hundreds of genome accessions available [1]. While among these accessions there are comparatively few that exhibit natural resistance to *T. myopaeformis*, some have been identified that are capable a successful defense response. Very little information is available regarding the molecular nature of resistance but the information that is available has been aided by the availability of the involved insect pest (*T. myopaeformis*) and plant genomes (*B. vulgaris*). Studies have identified a *B. vulgaris* gene, *BvSTI*, that is expressed during a successful defense response. The protein is related to Kunitz trypsin inhibitors (KTIs). KTIs, while understood to have defense roles, function to inhibit pathogen trypsins whose normal function is to facilitate infection. What is not understood in the *B. vulgaris*-*T. myopaeformis* pathosystem is the breadth and extent of those interactions which is now possible with the availability of host and insect pest genomes [2,3]. The value of the data becomes compounded when the knowledge is applied to other significant crop pests with global losses from plant insect pests estimated at $80B, annually [4]. For example, and more specifically, pathogens account for losses of 21.5% in wheat, 30.3% in rice, 22.6% in maize, 17.2% in potato, and 21.4% in soybean, five of the most significant global crops [5]. Consequently, the methods and information generated here aids comparative studies to other crops.

The data has been deposited in public databases or is available as supplemental data from the provided sites, available freely for use, with the extracted data provided here (**Supplemental Data 1**).

Data has been generated here to be used scientifically. The data provided here improves the current annotations in the host *B. vulgaris* and its hundreds of sequenced genomes, and an insect pest species (*T. myopaeformis*). The work expands on knowledge of the *KTI* and *Trp* gene families, while broadening that understanding to their inherent structural variation in ecological varieties not yet identified. Importantly, *B. vulgaris* is not native to North America. Therefore, the information generated here can be applied to the native plant species and communities in which *T. myopaeformis* naturally has existed and may allow for the identification of additional sources of resistance from those native species that could be applied to sugar beet cultivation. The annotation of the *Bv*KTIs and *Tm*Trp proteins presented here permits target identification for molecular study and manipulation to benefit crop improvement. The types of manipulation include suppression or perturbation, or enhancement through overexpression, RNA interference (RNAi), mutagenesis, clustered regularly interspaced short palindromic repeats (CRISPR)/CRISPR-associated protein 9 (Cas9) (CRISPR/Cas9)-mediated gene editing, base editing, or technology-based traditional breeding ([[6], [7], [8], [9], [10], [11], [12], [13], [14], [15], [16]]). It may also permit the development of pheromones and/or traps/small molecule inhibitors/elicitors to manage *T. myopaeformis*. Significantly, the end goal is to provide the scientific community and stakeholders with more new data so that the information can be used in its broadest context in ways that, otherwise, would not exist. The 3-dimensional protein-protein interaction maps are developed from the conceptually translated genome of the host, *B. vulgaris*, and its most significant pathogen, *T. myopaeformis*. The analysis shows for the first time that the *T. myopaeformis* Trps may have varying capacity to be bound by host (*B. vulgaris*) KTIs which may relate to insect pest success. The analysis relies on the AlphaFold deep learning program platform that predicts 3-dimensional protein conformation and interactions [17]. It is understood that the protein-protein interactions generated in AlphaFold are predictive and require additional *in vitro* and/or *in vivo* confirmation. However, studies show that not only do the AlphaFold predictions reflect *in vivo* interactions with high confidence, as then demonstrated by *in vitro* or *in vivo* studies, but enhance the determination of biological interactions not provided by other gold standard demonstrative methods [[18], [19], [20], [21], [22], [23], [24]]. The AlphaFold-predicted interactions can predict proven biological interactions, even across entire genomes and across kingdoms [[25], [26], [27], [28]]. However, it is important to accept the AlphaFold-based results are predictive and not scientific justification/evidence. Consequently, the results presented here would be enhanced by the aforementioned *in vitro* and *in vivo* confirmatory methods and would be a next step in determining their true *in vivo* function.

## 2 Background

SB is an important crop and one of only two plants, globally, from which sugar is widely produced, accounting for 35% of global raw sugar with an annual farm value in the U.S. of $1B alone [29,30]. While numerous pathogens detrimentally affect SB cultivation, the SBRM is among the most devastating with average yield losses of 42% while localized losses can be 100% [29,30]. Environment, banning of pesticides, and public perception exacerbate the problems related to *T. myopaeformis* control. Furthermore, the scarcity of genetic knowledge for *T. myopaeformis* impedes its agricultural mitigation [29]. The *de novo* sequenced and assembled *T. myopaeformis* draft genome, and annotation, are aiding its control [[2], [31], [32], [33], [34]].

## 3 Data Description

**Fig. 1:**
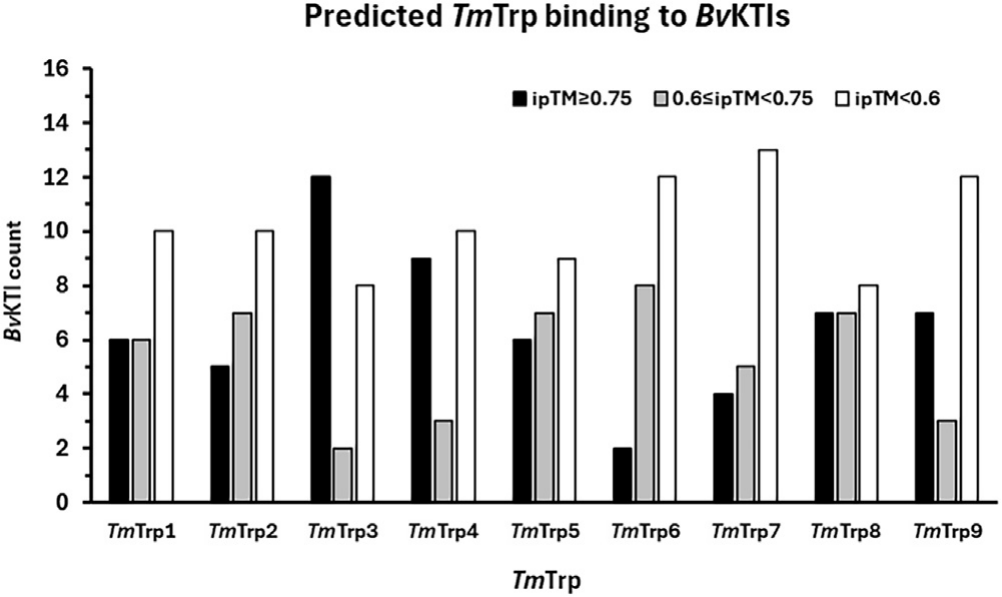
Numbers of *Bv*KTIs that are predicted to bind the *Tm*Trps.

**Fig. 2:**
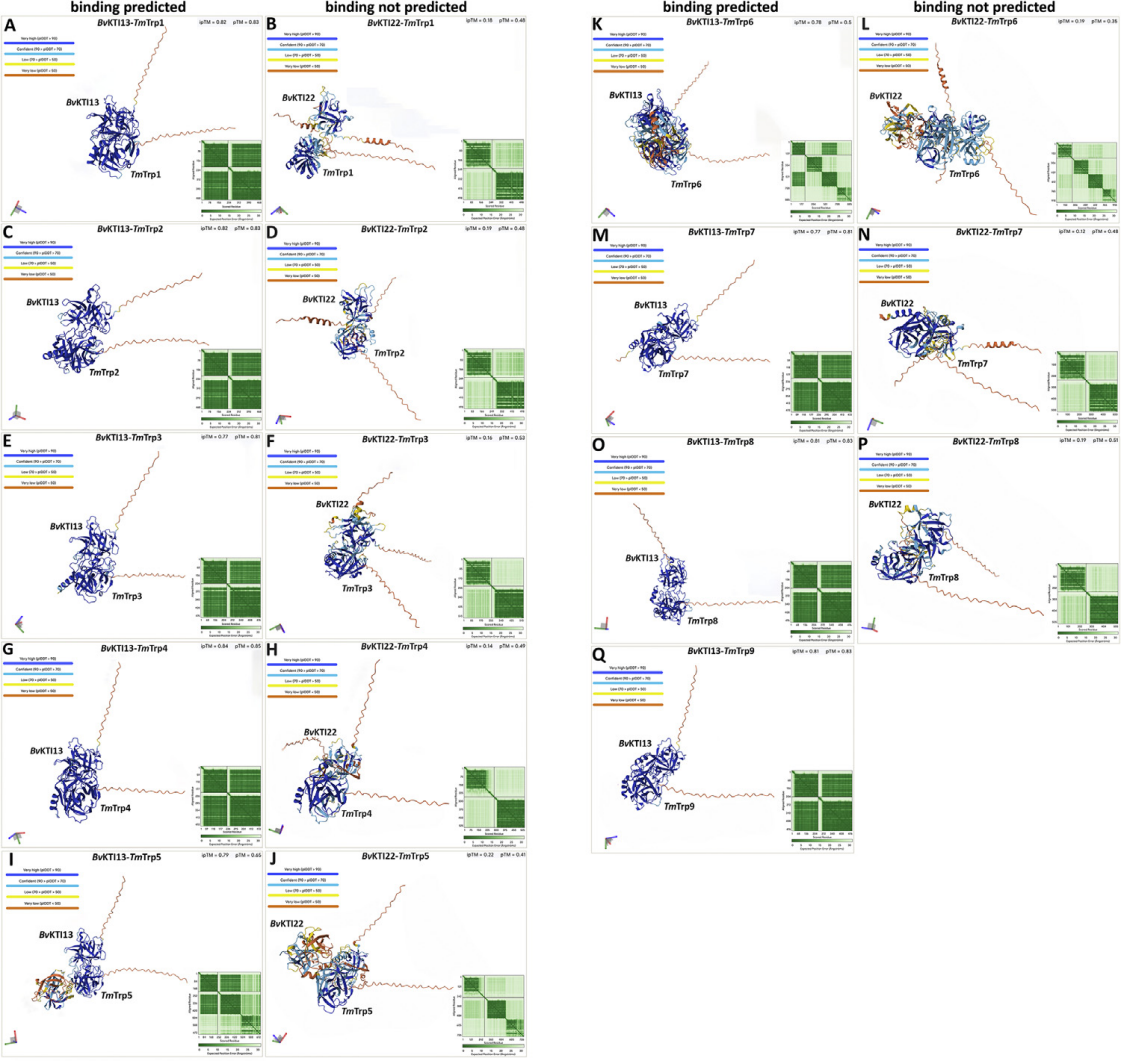
AlphaFold analysis. *Bv*KTI13 and *Bv*KTI22 are shown in their relationship to each of the 9 *Tm*Trps. **A.***Bv*KTI13-*Tm*Trp1; **B.***Bv*KTI22-*Tm*Trp1; **C.***Bv*KTI13-*Tm*Trp2; **D.***Bv*KTI22-*Tm*Trp2; **E.***Bv*KTI13-*Tm*Trp3; **F.***Bv*KTI22-*Tm*Trp3; **G.***Bv*KTI13-*Tm*Trp4; **H.***Bv*KTI22-*Tm*Trp4; **I.***Bv*KTI13-*Tm*Trp5; **J.***Bv*KTI22-*Tm*Trp5; **K.***Bv*KTI13-*Tm*Trp6; **L.***Bv*KTI22-*Tm*Trp6; **M.***Bv*KTI13-*Tm*Trp7; **N.***Bv*KTI22-*Tm*Trp7; **O.***Bv*KTI13-*Tm*Trp8; **P.***Bv*KTI22-*Tm*Trp8; **Q.***Bv*KTI13-*Tm*Trp9; **R.***Bv*KTI22-*Tm*Trp9. Legend: (*Tm*Trp-g7808 [*Tm*Trp1], *Tm*Trp-g3594 [*Tm*Trp2], *Tm*Trp-g23695 [*Tm*Trp3], *Tm*Trp-g23693 [*Tm*Trp4], *Tm*Trp-g3592 [*Tm*Trp5], *Tm*Trp-g7809 [*Tm*Trp6], *Tm*Trp-g23699 [*Tm*Trp7], *Tm*Trp-g23186 [*Tm*Trp8], and *Tm*Trp-g18287 [*Tm*Trp9]). Both the graphical view and predicted aligned error (PAE) images that indicates confidence in both the residue positioning and the overall structural model are shown. **A-I:** Representative *Bv*KTI13 that is predicted to bind each of the 9 *Tm*Trps. **J-R:** The representative *Bv*KTI22 that is predicted to not bind any of the 9 *Tm*Trps. The plDDT quantifies the confidence in the local structure of a protein by measuring the distance difference between the predicted and experimental structures. >90, very high; >90 pLDDT >70, confident; 70 > plDDT >, low; < 50 plDDT, very low. Importantly, the relationship between ipTM and piDDT in protein folding is that ipTM scores evaluate the accuracy of the relative positions of subunits in a protein-protein complex, while plDDT scores assess the overall accuracy of the predicted structure.

Infection of *T. myopaeformis*-resistant varieties of *B. vulgaris* were performed, leading to the identification of the increased relative transcript abundance (RTA) of the *B. vulgaris* protease inhibitor, DV501688 during a resistant reaction [35]. DV501688 was sequenced and annotated as *Bv*STI, prior to the availability of the sequenced *B. vulgaris* genome [35]. A BLASTp analysis of the *B. vulgaris* proteome identified 21 additional homologs annotated as Kunitz trypsin inhibitors (KTIs), *Bv*KTIs. The function of host KTIs is to inhibit pathogen Trps whose function is to facilitate infection. Thus, proteome search queries were performed here to identify Trps from the conceptually translated *T. myopaeformis* genome in order to perform predictive host KTI-pathogen Trp interaction analyses as a first step toward downstream *in vitro* and *in vivo* confirmatory studies. To accomplish this goal, BLASTp searches of the *T. myopaeformis* conceptually translated genome were performed by using the dipteran *Bactrocera oleae* Trp XP_014094233. The analysis identified 9 *T. myopaeformis* Trps (i.e. *Tm*Trp-g7808 [*Tm*Trp1], *Tm*Trp-g3594 [*Tm*Trp2], *Tm*Trp-g23695 [*Tm*Trp3], *Tm*Trp-g23693 [*Tm*Trp4], *Tm*Trp-g3592 [*Tm*Trp5], *Tm*Trp-g7809 [*Tm*Trp6], *Tm*Trp-g23699 [*Tm*Trp7], *Tm*Trp-g23186 [*Tm*Trp8], and *Tm*Trp-g18287 [*Tm*Trp9]) from the publicly available annotated conceptually translated genome of *T. myopaeformis*, BioSample accession: SAMN37733483, BioProject ID PRJNA1026092 (**Supplemental Fig. 1**). The data are available at the URL: https://www.ncbi.nlm.nih.gov/bioproject/PRJNA1026092. A BLASTp query of the conceptually translated *B. vulgaris* genome led to the identification of the 22 *Bv*KTI homologs (**Supplemental Fig. 1**. Deep learning predictive analyses using AlphaFold led to the identification of *Bv*KTIs that are predicted to dock to the *Tm*Trps in the predicted binding loop region located between β-strands 4 and 5 of KTI proteins (Table 1; **Supplemental Table 1**) [36]. The scope of predicted binding is shown graphically (Fig. 1). Representative analyses of *Bv*KTI13 which is predicted to bind all 9 *Tm*Trps, and *Bv*KTI22 which is predicted to not bind any *Tm*Trps, are shown (Fig. 2). The predicted binding is within the range demonstrated to be actual binding in *in vivo* studies, suggesting the predictions may represent *in vivo* function [37]. However, it is important to accept the AlphaFold-based results are predictive and not scientific justification/evidence and would be enhanced by the *in vitro* and *in vivo* confirmatory methods to determine their true *in vivo* function.

**Table 1:** Protein-protein interaction and trypsin cleavage.

The ipTM, derived from the predicted template modelling (pTM) score, which evaluates the overall accuracy of the predicted structure, measures interface quality between two proteins, showing how well the predicted structure aligns with the actual structure with values ≥0.75 treated as high confidence (red), values between 0.60 and 0.75 treated as intermediate confidence (blue), and values <0.60 considered low confidence (black) [38]. Yellow block, the interaction between *Bv*KTI13 and *Tm*Trps highlighting the predicted binding. Red block, the interaction between *Bv*KTI22 and *Tm*Trps highlighting they are not predicted to interact.

## 4 Experimental Design, Materials and Methods

The *T. myopaeformis* genome (https://www.ncbi.nlm.nih.gov/bioproject/PRJNA1026092) was *de novo* sequenced [2]. The sequence was archived at Genbank [39]. For the analysis presented here, the *T. myopaeformis* genome sequence was obtained from NCBI [2]. The genome assembly of Alkharouf et al. [2] used the pipeline Flye, version 2.9.2, to assemble the PacBio HiFi DNA reads using default values, except for setting the –asm-coverage argument to 50, to reduce memory consumption. The gene finding tool AUGUSTUS 3.5.0 was used for gene prediction analysis in these contigs with the complete gene option enabled and default set for the rest of the parameters [2,40]. *Drosophila melanogaster* is the most closely related genetic model most closely related, phylogenetically, to *T. myopaeformis* so it was used as the reference species to *T. myopaeformis* for TmSBRM_v1.0 assembly and annotation. The identified genes were used to perform a blast against the non-redundant (NR) database to predict genes. For generating the gene functional annotation, the predicted genes were functionally annotated using Blast2GO 6.0 using default values [41]. The gene model was BLASTed as BLASTp against the NCBI NR protein database. InterproScan 5.67-99.0 was used under default values for domain finding [42]. Then GO mapping and annotation was performed under default values using GeneOntology 2024-03-28 [43]. BLASTp of the *T. myopaeformis* proteome used the *Bactrocera oleae* Trp XP_014094233 in default settings to identify its Trps. BLASTp queries were done in the *B. vulgaris* RefBeet1.1 and RefBeet3.0 proteomes at https://bvseq.boku.ac.at/ set on default using DV501688 (Bv4_081010_nnrr.t1) *Bv*STI (*Bv*KTI1). The *B. vulgaris* proteome BLASTp was done in https://bvseq.boku.ac.at/Genome/ [3,44]. The 9 *T. myopaeformis* Trps identified from the conceptually translated TmSBRM_v1.0 genome are *Tm*Trp-g7808 [*Tm*Trp1], *Tm*Trp-g3594 [*Tm*Trp2], *Tm*Trp-g23695 [*Tm*Trp3], *Tm*Trp-g23693 [*Tm*Trp4], *Tm*Trp-g3592 [*Tm*Trp5], *Tm*Trp-g7809 [*Tm*Trp6], *Tm*Trp-g23699 [*Tm*Trp7], *Tm*Trp-g23186 [*Tm*Trp8], and *Tm*Trp-g18287 [*Tm*Trp9].

Protein–protein interaction predictions were performed using the AlphaFold-Multimer implementation available through the AlphaFold Server. The ipTM was used as an indicator of predicted global reliability, with values ≥0.75 treated as high confidence, values between 0.60 and 0.74 treated as intermediate confidence, and values <0.60 considered low confidence [38]. The bins varied comparatively little from the values (ipTM > 0.8 representing confident high-quality predictions; 0.6 ≤ ipTM ≤ 0.8 representing possible prediction; ipTM < 0.6 representing failed prediction) suggested by AlphaFold (https://www.ebi.ac.uk/training/online/courses/alphafold/inputs-and-outputs/evaluating-alphafolds-predicted-structures-using-confidence-scores/confidence-scores-in-alphafold-multimer/). Amino acid sequences of the candidate proteins were provided in FASTA format and submitted as a multimeric complex to enable structural modelling of potential interactions. The server generated predicted complex structures using deep learning–based modelling and multiple sequence alignment information. Model confidence and interaction reliability were evaluated using the predicted Local Distance Difference Test (predicted Local Distance Difference Test [pLDDT]) and the ipTM provided in the output. Trp cleavage prediction was performed by using PeptideCutter [45]. PeptideCutter was set at a lowest predicted cleavage displayed option of 100% [45]. Predicted protein interaction visualization was performed in default settings using AlphaFold [17,46].

## Limitations

The predicted *B. vulgaris* KTI-*T. myopaeformis* protein-protein interactions, the result of the AlphaFold analysis, are bioinformatic in nature. Consequently, they are not necessarily presenting actual *in vivo* interactions. However, the predicted binding is within the range demonstrated to be actual binding in *in vivo* studies [37]. Additional complications may result from sequence variation found in environmental populations of *B. vulgaris* and *T. myopaeformis*. However, those limitations are due to the organism’s populations and yet to be identified protein sequence variation and not AlphaFold. However, AlphaFold-based results are predictive and not scientific justification/evidence and would be enhanced by *in vitro* and *in vivo* confirmatory methods to determine their true *in vivo* function.

## Ethics Statement

The authors have read and follow the ethical requirements for publication in Data in Brief and confirming that the current work does not involve human subjects, animal experiments, or any data collected from social media platforms.

## CRediT authorship contribution statement

**Yerang Lee:** Methodology, Software, Validation, Formal analysis, Investigation, Resources, Data curation, Writing – original draft. **Sidra S. Rahim:** Methodology, Software, Validation, Formal analysis, Investigation, Resources, Data curation, Writing – original draft. **Nadim W. Alkharouf:** Methodology, Software, Validation, Formal analysis, Investigation, Resources, Data curation, Writing – original draft. **Chenggen Chu:** Investigation, Resources. **Vincent P. Klink:** Conceptualization, Methodology, Resources, Visualization, Supervision, Project administration, Funding acquisition, Writing – original draft.

## Data Availability

NCBITetanops myopaeformis genome (Reference data)

## Appendix Supplementary materials

**Supplemental Fig. 1.** The *Bv*KTI and *Tm*Trp accessions. Image, application 1

**Supplemental Table 1.** Predicted *Bv*KTI trypsin cleavage. Image, application 2

**Supplemental Data 1**. The *Bv*KTI and *Tm*Trp protein sequences have been extracted from conceptually translated genomes of *B. vulgaris* RefBeet1.1 proteome at https://bvseq.boku.ac.at/ set on default queried with DV501688 (Bv4_081010_nnrr.t1) *Bv*STI (*Bv*KTI1). The *T. myopaeformis* sequences were identified in NCBI, BioSample accession: SAMN37733483, BioProject ID PRJNA1026092 available at the URL: https://www.ncbi.nlm.nih.gov/bioproject/PRJNA1026092. Image, application 3
