# Supplementary material for: Predicted protein-protein interactions between sugar beet root maggot trypsins and sugar beet Kunitz trypsin inhibitors using deep learning
Source: Data Brief. 2026 Jun 27;67:113020. doi: 10.1016/j.dib.2026.113020 (PMC13342942; doi:10.1016/j.dib.2026.113020)
Supplement: Supplementary file 1 — Supplemental Fig. 1. The BvKTI and TmTrp accessions. [file mmc1.docx]

***Beta vulgaris* KTIs**

BvKTI1

>Bv4_081010_nnrr.t1 length=198 cDNAcoverage=100%

MASIFLKSTTTALLLIFSTLCIATAVVIQDTDGDALRNGGQYYIIPVSAGFQGGLTLKSKADNSPCPLYITRDKVETSRGIPVTIASPYRIAIITSSIPIGIVFTNTPNVCMQPLGWQVVADEKTGQSYVATGGNGFGFNPTESFDIQQIEGNNNVYKIRFAGESEVGFFEKDGLLGITNEIPLPVVFQKAFDVLAMV

BvKTI2

>Bv_06500_pdgi.t1 length=198 cDNAcoverage=100%

MASIFLKSTTTLILIFSALCIATAVILDTDGDALRNGGQYYIIPRSLGFGGGLTLNSKAGNTPCPLYITRENDETSPGIAVTIASPARIGIITSSLPISIVFNDIPNICMQPLWWQVIPDGTTGQSYVATGGSGIPFNPTETFSIEPIEGNIYKIRNGVGSDQARDVGFFENDGLLGITNDIPLPVVFRKAFDVLAMV

BvKTI3

>Bv_06520_zcfr.t1 length=199 cDNAcoverage=100%

MASIFLKSTTTVLLLISALSIATAFILDIDGDPLYNGGQYYIIPQSLGFGGGLTLKSKADSLPCPFYIGRENDETSHGMPVTIASPFKIGIITPSLPISIVFKGDGNPNICMQPLGWQVIADATTGQSYIATGGSGGAFNPTTTFSIEQIGININMYKIRNAGGSDVGFFEKDGLLGLTDDIPLHVVFRKAFDVLSMKV

BvKTI4

>Bv3u_069470_mnqs.t1 length=205 cDNAcoverage=100%

MASIFLKQTISLISLTLLLIFSALSISTAFVLDTDGEPLVNGGQYYIIPQSAGIMGRGLTLISKPDASPCPLHITRDKDETSLGIPATITSPFKVEFITDSVPINIVFKDTPNICVQSLAWQMIPDETTGQFYVATGGSGFLPTESFMVVKIGDKNVYKMRYIGGFDEKLYIGIFEKDGLLGSTNEIPLPVVFKKTSFDVLRMTN

BvKTI5

>Bv_06530_ieoe.t1 length=162 cDNAcoverage=100%

MQYYIIPQSLGFGGGLTLKSKAGTSPCPLYIGRENDETSPGIPVRISSPFMIATITPSLPINLVFKDTPSICMQPLGWQVIPDGTTGQSYVATGGSSIPFNPTETFGIEQIGDNNVYKISNRGTFGEARYVGFFEKDGLLGITNDIPLPVVFRKAFDVLSKV

BvKTI6

>Bv_06540_xxgs.t1 length=74 cDNAcoverage=63.6%

MNPEIRWNQIECPCQPLGTIDCGYYVCRYMLETVQLRRLLISTIKALVQTILQRKLMNSRSCGLHMSEINMKHK

BvKTI7

>Bv_06490_cgoi.t1 length=204 cDNAcoverage=100%

MGSIFLKSTTTLLLIFSTLSIATAFILDTDGEPLLNSRKYYILPQSLGFGGGLTRTTKNTDLPCPYYITRDNDETSNGMPLIISSPFRILYTPLSSPVYIAFEEMITVCIQSMGWRLIPDDSTGRSYVGTGASGFDLTQRFTIEHAGSEFENNVYKIRYIGESGEGRDVGFFKEDGLLGITDDIPLTVMFKKAFNVLEETTSKM

BvKTI8

>Bv_06600_jufw.t1 length=209 cDNAcoverage=100%

MASIFLKSTITLLLIFSTLSVAIAVVLDTDGEPLFNQVNNSRYYILPQTIGIGGGLTRTTKNPELPCPYYITRDNDETSSGMPLYISSPLKILFIPLSSPVRISFEEMPTICIQESMGWRVISDDSTGRLYVSTGAGRGPGRFTIEQAESESSNNVYKIRYIGALDSEAGAGDLGFFKEDGLLGITDDIPLTVVFKKAFDVPEETTTSM

BvKTI9

>Bv_06470_efpt.t1 length=201 cDNAcoverage=80%

MGSNFLKSITTPLIFSTLSIATAFILDTDGEPLFNSRKYYILPQSLGFGGGLIRTTKTTDLPCPYYITVDNDDTSYGMPLIISSPFRILYTPLSSPVYIAFEEMITVCIESMGWRLNPDNSTGRSTGASGFDLTQRFTIEHAGSEFENNVYKIKYIGASGEGRDVGFFDFNEDGLLGITDDIPLTVMFKKAIILEETTSRM

BvKTI10

>Bv_06550_muoq.t1 length=86 cDNAcoverage=100%

MQSLGWQVIADATTGQSYIATGGSGGAFNPTSTFSIEQIGVNMYKIRNAGGSDLGFFEKDGLLGLTDDIPLHVVFRKAFDVLSMKV

BvKTI11

>Bv_06580_frjx.t1 length=134 cDNAcoverage=60%

MGPTLPQSLGFGGGLTTTTKNTDLPCPYYVTRDKDETSNGMPLIISSPFRMLQIPLSSHVYIAFEQMITVCIQSMGWRLIPDDSTGRSYIGDSGEGRDVGFFKEDGLLGITDDIPLTVMFKKALNVLEETTSGM

BvKTI12

>Bv6_153420_udjq.t1 length=206 cDNAcoverage=100%

MTHVIISAATIFLFLYLSPLTSTADNTAVLDINGRPLQAGYNYYILPVIRGRGGGLTMASKNATELCPLYVAQEDHEVSNGLPLKFYPVNPKDKRISLSSDLNFVFDAATTCVQSTGWSLTIEMETGRRYVGTGGEIGNPGVETVDNWFKIEKDGSGKYDYKIVYCPGVCNFCKVMCGDVGVFIEKDGRRLLGFSDQPLLVMFKKA

BvKTI13

>Bv6_153430_sfyg.t1 length=208 cDNAcoverage=100%

MSHVVILSAATIFLFLCLSPLTSTAANIAVLDINGRPLQARSNYYILPVIRGRGGGLRMTPKNATQLCPLAYVAQEGSELANGLPLKFYPVNPKDKTISLSTDLNFVFDAATICVQSTQWRLAFDEVTGRRYVGFGGEIGNPGGNTVSNWFKIEKAETGKYDYKIVFCPGVCNFCKVACGEIGVFVEKDGRRLLGINNQPLLVMFKKA

BvKTI14

>Bv6_153400_criy.t1 length=262 cDNAcoverage=20%

MTQDIIISATTFLLLFLSPSWGNAADTSVLDTEGRPLRAKSRYYILPASQGQGGGITVSQKNQTTLCPLYVSQESQEIYLGLSVWFLPSKHNQRLIYISSDINILFNMVNICLQSAAWKLSIDHTTWRKYVATGGAIGNPGEETVSSWFKIEKVKSGSYEYDYNYKIMYCPNVCSFCMVMCGDVGVFVQDDGTSSIKDIQDLVLLRLGWWIKGWYEEFPYSAIDIQRTPSCLLWNGFAAVHPPIKSLSTPVIWNPPVVNHLK

BvKTI15

>Bv6_153410_hihh.t1 length=211 cDNAcoverage=100%

MTPHSILSILTILLFLLIAPLSTTTAAAKTTTVLDINGRPLKTDSTYYILPVSRGRGGGLAMAPKNATESCPLYVAQENHEVSNGLPLKFFPTNPNDHDKIPLGYDVNIVFDAATICVQPTGWMLAFDEASGTRYVGIGGTIGNPGVDTLSNWFAIEKAGSGLYDYKIRFCPAVCIFCTVMCGDVGVFIGEGGTRFLALTDRPLLVRFKKA

BvKTI16

>Bv3_066450_dhqp.t1 length=212 cDNAcoverage=66.7%

MATHFISSTLVLATFLLFVSPPAAVAQVINIFDMDAEPVQAGKLYYILPVVQRQGGGISTAPKNANESSCPLYVVQEKDTTSLGLAVTFNLALPNTTNVTFSADMNIVFGQAINCVESPVWTLALDEPTGRRYVALGGLGMARGPKAVNNWFRIERFFPRFHFDYKFVFCPTRVICPTCQNSCGDLGVFVRDDGTWVLGVGAPPLRIKFKKA

BvKTI17

>Bv6_153440_aich.t1 length=221 cDNAcoverage=100%

MSHLILQLSVTILLIFSLPTNIATKTTDDSLVLDVDGNPIEVGSAYYVRTELSKAGIGGGLVTASKPNHTQCPQYVAQLAYGFEGEIPVTFYPSSSSQKFIHISSDINIIFNTTSNVCSQGAWQLTPDASNGNLYLSTGGVIGNPGSLTTANWFKIAKSPYDPNFYQLEYCPDTKTVASPTGDIVCGAIDALDSTDDFLLMWLGLKTIRPDFFHWLSFVKA

BvKTI18

>Bv6_153590_kpiu.t1 length=228 cDNAcoverage=100%

MSHLHFIFSFFLLSLLLSPSITSAAESLLDTDGNPLSSSGSYYVLPVSWGAGGGLNIAAIQDHHTRCPYYSVVQSQDDRCGSNLGLPVTFTPSDLEEGQNITLATDFSIDFNIRPPHAPRFCDQPTTTWEMVSVDGPNNIGAQLGSFHHGGLTRGGGDDKQGSLFKMVKNKAYGYRLRYCPTNSSKNSPLKNVVCGDLAPVYDRRLGVRVLSLVDDSYRPFEIMFKKA

BvKTI19

>Bv6_153580_rnpy.t1 length=221 cDNAcoverage=100%

MISSAQLIYDTSGDIVTSSNAYYLMPVSRGSGGGFNIAAVQDYHTRCPYYSVVQSPHDRCGSYFGLPVKFSRSSTSTTADSNHQEVNVTLSTDFTINFSTYTPHAPRYCDQPTTMWNMVSRGGSGDDMSVELGMVHFGGLNHDDDDDDNDDDDDRQSSLFKIVKNKAYGYRLRFCPSNSNSSALKDVVCGDLAPVYDRRLGVRVLRLVDSKSPAMIMFHKA

BvKTI20

>Bv8_192190_tujs.t1 length=221 cDNAcoverage=100%

MKHLILFLASLYCAVTAAAITTITADDNSVYTVIGDPLLAGQDYYVVPYNYGDVGGGLTWKLKQELLAICPHYYVLQVEDNLNFGEPTAFFPSNSRQNQITLSNKLNILFSKVSPPASPPFVCPASSNVWKVVADDSTGIVYVELGGRKGRSRDTNSWFTIEELDEGGYQIKYCDSTKRKTASVCGGLGIVSLNGGRYLGVNATNPVAFAFETQQIFVKTA

BvKTI21

>Bv_41530_sisg.t1 length=235 cDNAcoverage=100%

MAHQFILQAIATILVTLLFLSCPITAGSPVLDLDGNPLEVGSQYYVLTAGWGAAGRGGLTDTFKPPSVCPSYVAQHRLWTNNGRPVTFYPSDSSQKQIIQGDEMNIAFGIIPLCRSTGIWRLTFDNETQVPYVATNGVIGNPGTFETFGNWFKIEKAFGDSSYKIVFCFVEPVPAPGNTNLVGRRRHCEQLDATQKGPNGDLSYLSLVPLDQNLPFFGYVFKKVDTSSATTTAFY

BvKTI22

>Bv6_153450_yxie.t1 length=239 cDNAcoverage=100%

MKHLFLPLATTTTLLLFLLLLLSTPPTNATDITVYDAIGEPLVAGQSYYLVPYEYIDVGGGGLTWKQKVKSEGNCAPYYVSQQNENTDFGSQITFFPQNTRQTEITTSTELNIVFDSYSPPYNCPGSSNVWRTALDRATQTHFIELAGRKGTRDVRTWFTIQELGPDLGGYKIRYCPRHRQADCGDLGIFPRANVIDTRWLCVNGSNPLAFMFESEELFLKNKARRSVPTYNLGTTYAE

***T. myopaeformis* trypsins**

TmTrp1

>g7808

MLLPTATLSSTAIGIYIFAALLPCYWGQNLTIDKRIIGGKPIQIQRAPFMVNLREDGRFVCGGTLLTRKCVLTAAHCVAGVSTSRLTIQGAVTRLTQSGQMSNVRSVFVPRAYSRRTSNYDVAILRLAKPITNRHARTVKLARRALRPGTRVSVFGWGRSQEDGKFSKSLRAATVRVLPHASCKSRYRNVAVLTRSMFCASMPGVRDSCSGDSGGPVVYRGVQYGIVSWGVGCARRQYPGVNTHIHVVRPWIDSIAAQQC

TmTrp2

>g3594

MLLPTATLSSTAIGIYIFAALLPCYWGQNLTIDKRIIGGKPIQIQRAPFMVNLREDGRFVCGGTLLTRKCVLTAAHCVAGVPTSRLTIQGAVTRLTQSGQMSNVRSVFVPRAYSRRTSNYDVAILRLAKPITNRHARTVKLARRALRPGTRVSVFGWGRSQEDGKFSKSLRAATVRVLPHASCKSRYRNVAVLTRSMFCASMPGVRDSCSGDSGGPVVYRGVQYGIVSWGVGCARRQYPGVNTHIHVVRPWIDSIAAQQC

TmTrp3

>g23695

MALAIANVAARAAGDKVTQTQRVPRLTDNRIVGGVEVDIARAPYQVSLRYKILFDPSYSYSHFCGGSIYSDTVVITAAHCIIGTVPSQFQIAAGTSNCCGGDGIIVPVKDIIMHQDYDPSTAKNDIAIMILAAPLPLNNFTIQAIALTESEVPEGATSIITGWGDTTPSGTPADKLQEVRVPIVSNAACNEDYKNIEVDDSMLCAGLRGVGGKDACQGDSGGPLVVNGKLAGVTSWGYGCALPQYPGVYAKVSHLRPWLLEKLEEYKINLN

TmTrp4

>g23693

MFKIALVGLLLVGIAHGATPTGDLEGRIVGGNDVSIVKHPYQVSVRFKSCNACAYVHECGGSIYNEDTIISAAHCLHHREPRDFVVVAGTDNRVGSDGVVTRIEKIVTHEKYNASITDNDVALLFLATPLPINHVTIERVSLTAETPGTGVKSVVIGWGATTEGGAYSQKLQEAEVAIISKEACQEAYNGGRTITEGMLCARVAGGGKDACKGDSGGPLLVKKELAGIVSWGIGCARPESPGVYADVAYYGEWLRLTITENSLFA

TmTrp5

>g3592

MPFPATDAIRAALVCLLVALASASHKFAALSDTRIVGGVETTISEVPYLVNIRRNGQFSCGGSLITTTCVLTAAHCVRGVTPSSLTIHAGSSRLSQSGQAEQAEKHYVSPFYSSTTLDMDVAIIKLANPLNGPNIATISLCGHSPESEQFVKISGWGITNEYSNAPPDQVRTTSVRVVAKDDCIRAYAGKALLTSTMFCATVAGERDSCSGDSGGPVVYDGRVCGIVSWGFGCARQEYPGVYTNVASRRRHVALAQPRIVGGVTTTIQAAPYLVNLRINGVFFCGGSLVTRQHVVTAAHCVKGKNVGAIVVTAGTSQLDGSGITRSVAKAFLPNKYRRDNENMDVAVLKLQSPINQGNVRPIDLCNTRLKVGNRLTVFGWGVTNENSKTSSNQYRFRARLTRTMFCASVPGSKDSCAGDSGGPAVFGGRLCGIVSFGVGCARRNYAGVYTSCCNRSLILFTLSCA

TmTrp6

>g7809

MSHLIVLVILAVSAIAASATQPPANRARRIVGGDLAVSTQIPYLVNIRKNGVFHCGGSLVTSRCVLTAAHCVRGGAPQDFVVRAGVTFLTDFQNGRRVDQIFTPDQYNNKTLEHDIAVFRLKVAISADRSMKPIRIADFVPKQGELVRVSGWGFTHENATHPPNQMRTVRVRVMQQEQCQALYADYRNVSETMFCASMPGYKDACMADSGGPAVAHGQLVGVVSWGKGHDCGRAESPGVYASLSTMRRIVGGVETTISEVPYLVNIRRNGQFSCGGSLITTTCVLTAAHCVRGVTPSSLTIHAGSSRLSQSGQAEQAEKHYVSPFYSSTTLDMDVAIIKLANPLNGPNIATISLCGHGPESEQFVKISGWGITNEYSNAPPDQVRTTSVRVVAKDDCIRAYAGKALLTSTMFCATVAGERDSCSGDSGGPVVYDGRVCGIVSWGFGCARQEYPGVYTNVASRRRHIALAQPRIVGGVTTTIQAAPYLVNLRINGVFFCGGSLVTRQHVVTAAHCVKGKNVGAIVVTAGTSQLDGSGITRSVAKAFLPNKYRRDNENMDVAVLKLKSPINQRNVRPIDLCNTRLKVGNRLTVFGWGVTNENSKTSSNQYRFRARLTRTMFCASVPGSKDSCAGDSGGPAVFGGRLCGIVSFGVGCARRNYAGVYTSCCNRSLILFTLSCA

TmTrp7

>g23699

MFRLAIVVCALLAAGGARADTHQIGMLPMPDGRIVGGSNADIRQYPHQISMRYKGRHRCGGSIYSANVIVSAAHCVFETDASLITIVAGSTLLSKKNVEIPVLKYIIHSSYKVFNNDYDVAILVLNGRLNFNQYIQPIALARERPATGTEVTVTGWGTLVENGNSPDHLQQVNVNVVDNSNCRKSYLVLLTKRMLCAGVDGGGQDACQGDSGGPLIVNNQLLGIVSWGVGCAQKHFPGVYASVPDLADWIEATVAANTLEFVQT

TmTrp8

>g23186

MTRLAVALLFVSASISAAVPLDAGATAAAAEPEKRIVGGMQTSIAKYPYQVSVRLDSSALIHICGGSIYAPRVIVTAAHCLKGRYASHIRVVAGSSTIADQSEQGVAAKKLIYHSGYSKKTHENDVGLIILAADLVWSDVVQPIPLAPEDPAAGTHATVTGWGKSDEEAKTLTNMLQEVDVKIVDTNVCHAQYTAKDYTITEQMVCAGVEAGGKDTCQGDSGGPMVTSGKLVGIVSWGIGCARSDFPGVYASVPYHADWITAQAQPYL

TmTrp9

>g18287

MTRLAVALLFVSASISAAVPLDAGATAAAAEPEKRIVGGMQTSIAKYPYQVSVRLDSSALIHICGGSIYAPRVIVTAAHCLKGRYASHIRVVAGSSTIADQSEQGVAAKKLIYHSGYSKKTHENDVGLIILAADLVWSDVVQPIPLAPEDPAAGTHATVTGWGKSDEEAKTLTNMLQEVDVKIVDTNVCHAQYTAKDYTITEQMVCAGVEAGGKDTCQGDSGGPMVTSGKLVGIVSWGIGCARSDFPGVYASVPYHADWITAQAQPYL
